# Evidence of Hypoxia Signaling and Endothelial Activation in Migraine: Relationships Between HIF-1α, VEGF-A, and Arginine Metabolism

**DOI:** 10.3390/biomedicines14071458

**Published:** 2026-06-27

**Authors:** Seyma Dumur, Mohammad Mahdi Bagheri Asl, Demet Aygun, Dildar Konukoglu, Hafize Uzun

**Affiliations:** 1 Department of Medical Biochemistry, Faculty of Medicine, Istanbul Atlas University, 34408 Istanbul, Turkey; hafize.uzun@atlas.edu.tr; 2 Department of Neurosurgery, Medical University of South Carolina, Charleston, 135 Cannon Street, Charleston, SC 29425, USA; mm.bagheriasl@gmail.com; 3 Department of Neurology, Faculty of Medicine, Istanbul Atlas University, 34203 Istanbul, Turkey; demetaygun@yahoo.com; 4 Department of Medical Biochemistry, Cerrahpasa Faculty of Medicine, Istanbul University-Cerrahpasa, 34098 Istanbul, Turkey

**Keywords:** migraine, chronic migraine, biomarkers, HIF-1α, VEGF-A, hypoxia, endothelial dysfunction, arginine metabolism, nitric oxide, ornithine

## Abstract

**Background/Objectives:** Migraine is a common neurovascular disorder associated with substantial disability. Increasing evidence suggests that hypoxia-related signaling, endothelial dysfunction, and nitric oxide metabolism contribute to its pathophysiology. This study investigated the relationships between hypoxia-inducible factor-1 alpha (HIF-1α), vascular endothelial growth factor A (VEGF-A), and arginine pathway metabolites in chronic migraine. **Methods:** In this observational study, fasting ethylenediaminetetraacetic acid (EDTA) plasma samples were obtained from 28 patients with chronic migraine and 28 healthy controls. Arginine, citrulline, and ornithine concentrations were quantified by liquid chromatography–tandem mass spectrometry, whereas HIF-1α and VEGF-A were measured using enzyme-linked immunosorbent assays. Group comparisons, receiver operating characteristic analyses, and Firth penalized logistic regression models were performed. **Results**: Patients with chronic migraine exhibited significantly higher VEGF-A and HIF-1α concentrations than controls (both FDR-adjusted *p* ≤ 0.001). VEGF-A demonstrated excellent discrimination of migraine status (AUC = 0.973), whereas HIF-1α showed good discriminatory performance (AUC = 0.794). The arginine-to-citrulline ratio was higher (FDR-adjusted *p* = 0.032) and ornithine concentrations were lower (FDR-adjusted *p* = 0.043) in migraine patients. In multivariable analyses, VEGF-A (OR = 14.46), HIF-1α (OR = 5.83), and ornithine (OR = 0.28) remained independently associated with migraine status. **Conclusions:** Chronic migraine was associated with elevated circulating HIF-1α and VEGF-A concentrations together with alterations in arginine metabolism. These exploratory findings suggest that hypoxia-responsive signaling, endothelial activation, and nitric oxide-related metabolic pathways may represent interconnected biological processes associated with chronic migraine. Larger longitudinal and externally validated studies are required to confirm these observations and clarify their potential clinical relevance.

## 1. Introduction

Migraine is a highly prevalent neurological disorder and one of the leading causes of years lived with disability worldwide, with a particularly high burden among young and middle-aged women [1,2]. Although migraine is clinically defined by recurrent headache attacks and associated symptoms such as nausea, photophobia, phonophobia, and, in some patients, aura, its biological basis extends beyond episodic pain perception alone. Current concepts describe migraine as a complex neurovascular disorder involving interactions among neuronal excitability, trigeminovascular activation, cortical spreading depolarization, neuroinflammation, vascular regulation, and metabolic vulnerability [3,4,5].

Disturbances in oxygen homeostasis may be relevant to migraine biology. Experimental and clinical observations indicate that hypoxia can provoke headache with migraine-like features, and high-altitude headache and acute mountain sickness frequently overlap phenotypically with migraine [6,7]. Hypoxia may also interact with cortical spreading depolarization, trigeminovascular signaling, oxidative stress, and inflammatory cascades, thereby providing a plausible mechanistic link between oxygen-sensitive pathways and migraine susceptibility [6,7,8]. Hypoxia-inducible factor-1 alpha (HIF-1α) is a central transcriptional regulator of cellular adaptation to reduced oxygen availability and modulates genes involved in angiogenesis, vascular remodeling, inflammation, and energy metabolism [9,10]. HIF-1α is a ubiquitously expressed intracellular transcription factor present in virtually all nucleated cells, while vascular endothelial growth factor A (VEGF-A) is a hypoxia-inducible secreted angiogenic factor produced by multiple cell types across different tissues [9,10,11,12].

VEGF-A is a major downstream mediator of hypoxia-related signaling and is a key regulator of angiogenesis, endothelial permeability, and vascular homeostasis [11,12]. Endothelial dysfunction has been repeatedly proposed as a contributor to migraine pathophysiology, particularly through impaired vascular reactivity, oxidative stress, altered nitric oxide signaling, and abnormal neurovascular coupling [13,14,15]. Previous biomarker studies have investigated vascular and inflammatory mediators in migraine, including VEGF-A, but the relationship between VEGF-A, hypoxia-related signaling, and metabolic pathways remains incompletely characterized [16,17].

Nitric oxide (NO) signaling represents another biologically relevant pathway in migraine. Nitroglycerin, an NO donor, can trigger genuine migraine attacks in susceptible individuals, and NO-mediated mechanisms are considered important in cranial vascular regulation and trigeminovascular nociceptive processing [18,19,20]. Arginine is the principal substrate for NO synthesis, whereas citrulline and ornithine are closely linked metabolites within arginine metabolism. Alterations in this pathway may therefore influence endothelial function, vascular tone, oxidative balance, and pain-related neurovascular signaling [21,22]. Although hypoxia-related signaling, endothelial dysfunction, and nitric oxide metabolism have individually been implicated in migraine pathophysiology, studies integrating these complementary biological domains within the same patient population remain limited.

We hypothesized that chronic migraine is associated with a distinct hypoxia–endothelial–metabolic biomarker profile characterized by increased HIF-1α and VEGF-A concentrations together with alterations in arginine pathway metabolism. Therefore, the present study aimed to perform an integrated evaluation of circulating HIF-1α, VEGF-A, arginine pathway metabolites, and derived metabolic indices in patients with chronic migraine and healthy controls, and to investigate their interrelationships and associations with migraine status.

## 2. Materials and Methods

### 2.1. Study Design and Participants

We conducted an observational cross-sectional study investigating hypoxia-related biomarkers, endothelial activation markers, and arginine pathway metabolites in patients with chronic migraine and healthy controls.

Adult patients fulfilling the International Classification of Headache Disorders, 3rd edition (ICHD-3) diagnostic criteria for chronic migraine [23] were consecutively recruited from the Neurology Outpatient Clinic. Healthy controls without a history of migraine, chronic headache disorders, or major neurological disease were enrolled for comparison.

Participant recruitment and sample collection were performed between 29 April 2026 and 29 May 2026. Demographic and clinical characteristics, including age, sex, body mass index (BMI), smoking status, migraine duration, monthly attack frequency, aura status, Migraine Disability Assessment Scale (MIDAS) scores, and Visual Analog Scale (VAS) pain scores, were recorded at enrollment.

The study protocol was approved by the Non-Interventional Scientific Research Ethics Committee of Istanbul Atlas University (Approval No. 04/43; 27 April 2026). All participants provided written informed consent before inclusion. The study was conducted in accordance with the Declaration of Helsinki and its subsequent amendments.

### 2.2. Outcomes

The primary outcome was the comparison of circulating hypoxia-related and endothelial biomarkers, including hypoxia-inducible factor-1 alpha (HIF-1α) and vascular endothelial growth factor A (VEGF-A), between patients with chronic migraine and healthy controls.

Secondary outcomes included comparisons of plasma arginine pathway metabolites (arginine, citrulline, and ornithine), evaluation of derived metabolic ratios, assessment of correlations between biomarkers and migraine-related clinical variables, and determination of the discriminatory performance of individual biomarkers for migraine status.

### 2.3. Blood Collection and Sample Processing

Following an overnight fast, peripheral venous blood samples were collected between 08:00 and 10:00 a.m. into EDTA-containing tubes. Samples were centrifuged promptly after collection, and plasma aliquots were separated and stored at −80 °C until analysis. Repeated freeze–thaw cycles were avoided.

### 2.4. Measurement of HIF-1α and VEGF-A

Plasma concentrations of HIF-1α and VEGF-A were measured using commercially available sandwich enzyme-linked immunosorbent assay (ELISA) kits (Elabscience, Houston, TX, USA) according to the manufacturer’s instructions.

HIF-1α concentrations were determined using the Human HIF-1α ELISA Kit (Human HIF-1α ELISA Kit (Catalog No. E-EL-H6066; Elabscience, Wuhan, China)), which has a reported analytical sensitivity of 37.5 pg/mL and a detection range of 62.5–4000 pg/mL. VEGF-A concentrations were determined using the Human VEGF-A ELISA Kit (Catalog No. E-EL-H0111; Elabscience, Wuhan, China), with a reported analytical sensitivity of 18.75 pg/mL and a detection range of 31.25–2000 pg/mL. Assays were performed according to the manufacturer’s protocol, and absorbance was measured at 450 nm. Biomarker concentrations were calculated from standard calibration curves.

### 2.5. LC–MS/MS Analysis of Arginine Pathway Metabolites

Plasma arginine, citrulline, and ornithine concentrations were quantified using liquid chromatography–tandem mass spectrometry (LC–MS/MS) on a Thermo Scientific platform. Chromatographic separation was achieved using an Accucore HILIC analytical column (3 mm internal diameter; Thermo Scientific, Waltham, MA, USA). The mobile phase consisted of acetonitrile and 100 mM ammonium formate buffer (pH 3), and analytes were detected in positive electrospray ionization mode using multiple reaction monitoring (MRM).

Quantification was performed using isotope-labeled internal standards and multi-level plasma calibrators. Internal quality-control materials were included in each analytical run. Method validation included assessment of linearity, recovery, analytical sensitivity, and precision. Reported intra-assay and inter-assay coefficients of variation were below 6.2% and 5.5%, respectively.

To evaluate pathway activity, the arginine-to-citrulline ratio and arginine-to-ornithine ratio were calculated and included in subsequent analyses.

### 2.6. Statistical Analysis

Statistical analyses were performed using R software (version 4.5.2; R Foundation for Statistical Computing, Vienna, Austria).

Data distributions were assessed visually and using the Shapiro–Wilk test. Continuous variables are presented as mean ± standard deviation (SD) or median with interquartile range (IQR), as appropriate, whereas categorical variables are presented as frequencies and percentages.

Between-group comparisons were performed using independent-samples *t*-tests or Mann–Whitney U tests according to data distribution. To account for multiple testing, *p*-values were adjusted using the Benjamini–Hochberg false discovery rate (FDR) procedure.

Associations between biomarkers and clinical variables, including MIDAS scores, VAS scores, migraine duration, and monthly attack frequency, were evaluated using Spearman’s rank correlation analysis.

Receiver operating characteristic (ROC) analyses were performed to assess the discriminatory ability of individual biomarkers. Areas under the curve (AUCs) and corresponding 95% confidence intervals (CIs) were calculated.

Independent associations between biomarkers and migraine status were evaluated using multivariable Firth penalized logistic regression models adjusted for age, sex, BMI, smoking status, and serum creatinine concentrations.

Exploratory principal component analysis (PCA) and biomarker correlation analyses were performed to investigate clustering patterns and biological relationships among the measured variables.

All statistical tests were two-sided, and *p*-values < 0.05 were considered statistically significant.

## 3. Results

### 3.1. Study Population and Baseline Characteristics

A total of 56 participants were included in the study, comprising 28 patients with chronic migraine and 28 healthy controls. Overall, 39 participants (69.6%) were female and 17 (30.4%) were male. The median age of the study population was 40.0 years (IQR, 30.5–48.0), and the median body mass index (BMI) was 21.0 kg/m^2^ (IQR, 19.0–23.0).

Baseline demographic and clinical characteristics are presented in Table 1. Age distribution was comparable between migraine and control groups (40.0 years [IQR, 29.5–48.0] vs. 39.5 years [IQR, 31.5–48.5], *p* > 0.9). Although female participants were more frequent among patients with migraine (78.6% vs. 60.7%), the difference was not statistically significant (*p* = 0.245). Smoking was significantly more common in the migraine group than in controls (50.0% vs. 17.9%, *p* = 0.023). Patients with migraine also had a significantly higher BMI than healthy controls (23.0 kg/m^2^ [IQR, 21.0–24.0] vs. 19.0 kg/m^2^ [IQR, 18.3–20.5], *p* < 0.001). Urea and creatinine concentrations did not differ significantly between groups.

Among patients with chronic migraine, the median disease duration was 11.0 months (IQR, 6.0–21.8), and the median monthly migraine frequency was 11.0 attacks (IQR, 6.0–14.3). The median MIDAS grade was 3.0 (IQR, 2.0–3.0), and the median VAS pain score was 8.0 (IQR, 7.0–9.0).

### 3.2. Comparison of Biomarkers Between Groups

**Figure 1:**
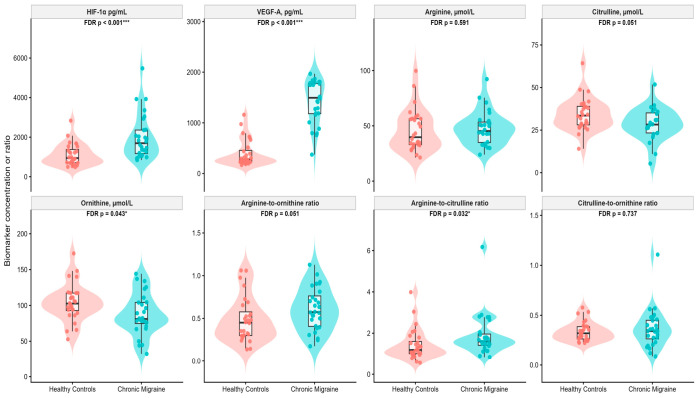
Distribution of hypoxia-related, endothelial, and arginine pathway biomarkers in patients with chronic migraine and healthy controls. Violin plots showing the distribution of circulating HIF-1α, VEGF-A, arginine, citrulline, ornithine, arginine-to-ornithine ratio, arginine-to-citrulline ratio, and citrulline-to-ornithine ratio in patients with chronic migraine and healthy controls. Violin shapes represent kernel density distributions, overlaid points indicate individual observations, and embedded boxplots show median values and interquartile ranges. Significant between-group differences were observed for VEGF-A, HIF-1α, ornithine, and the arginine-to-citrulline ratio after false discovery rate (FDR) correction. * *p* < 0.05, *** *p* < 0.001.

Comparisons of hypoxia-related, endothelial, and arginine pathway biomarkers between patients with chronic migraine and healthy controls are summarized in Table 2 and Figure 1.

VEGF-A concentrations were significantly higher in patients with migraine than in controls (1494.00 pg/mL [IQR, 1181.50–1776.71] vs. 279.41 pg/mL [IQR, 226.29–456.71]). The median difference was 1214.59 pg/mL, corresponding to a Hodges–Lehmann estimate of 1076.66 pg/mL (95% CI, 873.33–1290.83). This difference remained significant after false discovery rate (FDR) correction (rank-biserial r = 0.946, FDR-adjusted *p* < 0.001).

HIF-1α concentrations were also significantly higher in the migraine group (1693.96 pg/mL [IQR, 1172.74–2360.00]) than in controls (942.08 pg/mL [IQR, 710.94–1374.62]). The median difference was 751.88 pg/mL, with a Hodges–Lehmann estimate of 630.19 pg/mL (95% CI, 307.54–1083.02). This association remained significant following FDR correction (rank-biserial r = 0.588, FDR-adjusted *p* = 0.001).

Among arginine pathway biomarkers, the arginine-to-citrulline ratio was significantly higher in patients with migraine than in controls (1.60 [IQR, 1.42–1.96] vs. 1.19 [IQR, 1.01–1.60]). The median difference was 0.41, with a Hodges–Lehmann estimate of 0.41 (95% CI, 0.09–0.67). This difference remained significant after FDR correction (rank-biserial r = 0.393, FDR-adjusted *p* = 0.032).

Conversely, ornithine concentrations were significantly lower in the migraine group (81.10 µmol/L [IQR, 74.82–104.12]) than in controls (102.60 µmol/L [IQR, 92.87–117.22]). The median difference was −21.50 µmol/L, corresponding to a Hodges–Lehmann estimate of −16.88 µmol/L (95% CI, −29.96 to −4.07). This association remained significant after FDR correction (rank-biserial r = −0.360, FDR-adjusted *p* = 0.043).

Although citrulline concentrations and the arginine-to-ornithine ratio showed nominally significant between-group differences, neither remained significant after correction for multiple testing (both FDR-adjusted *p* = 0.051). Arginine concentrations and the citrulline-to-ornithine ratio did not differ significantly between groups Detailed distributional characteristics and normality assessments for all biomarkers are provided in Supplementary Table S1 and complementary geometric mean summaries are provided in Supplementary Table S2.

### 3.3. Correlation Structure Among Biomarkers

**Figure 2:**
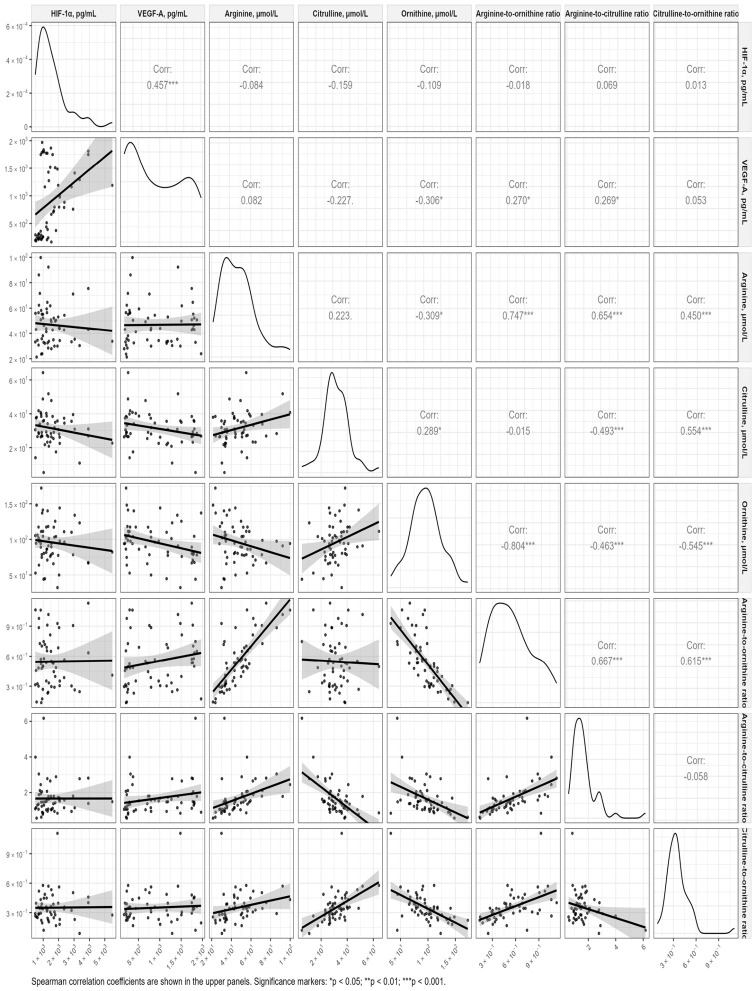
Correlation Structure among Hypoxia-Related, Endothelial, and Arginine Pathway Biomarkers. Spearman correlation matrix illustrating pairwise relationships among HIF-1α, VEGF-A, arginine, citrulline, ornithine, and derived metabolic ratios in the study population. Density distributions are shown along the diagonal, scatterplots with fitted trend lines are presented in the lower triangle, and Spearman correlation coefficients are displayed in the upper triangle. Significant correlations after Benjamini–Hochberg false discovery rate (FDR) correction are indicated by asterisks. A moderate positive correlation was observed between HIF-1α and VEGF-A, while ornithine demonstrated inverse associations with VEGF-A and arginine. * FDR-adjusted *p* < 0.05; *** FDR-adjusted *p* < 0.001.

Spearman correlation analysis revealed expected relationships among arginine pathway metabolites and their derived indices (Figure 2). The strongest association was observed between ornithine and the arginine-to-ornithine ratio (ρ = −0.804, FDR-adjusted *p* < 0.001). Additional strong positive correlations were observed between arginine and the arginine-to-ornithine ratio (ρ = 0.747, FDR-adjusted *p* < 0.001), arginine-to-ornithine ratio and arginine-to-citrulline ratio (ρ = 0.667, FDR-adjusted *p* < 0.001), and arginine and the arginine-to-citrulline ratio (ρ = 0.654, FDR-adjusted *p* < 0.001).

Citrulline was positively associated with the citrulline-to-ornithine ratio (ρ = 0.554, FDR-adjusted *p* < 0.001) and inversely associated with the arginine-to-citrulline ratio (ρ = −0.493, FDR-adjusted *p* < 0.001).

Among the hypoxia-related biomarkers, HIF-1α and VEGF-A demonstrated a moderate positive correlation (ρ = 0.457, FDR-adjusted *p* = 0.001). VEGF-A was inversely correlated with ornithine concentrations (ρ = −0.306), while arginine was also inversely correlated with ornithine (ρ = −0.309); both associations remained significant after FDR correction.

### 3.4. Receiver Operating Characteristic Analysis

**Figure 3:**
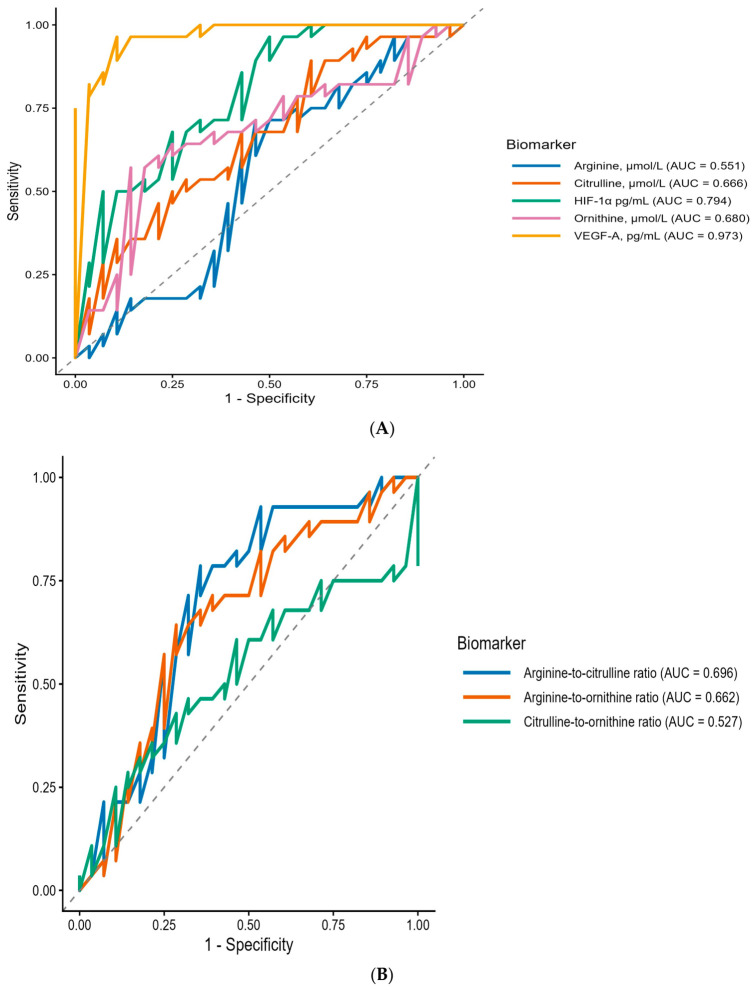
Receiver operating characteristic (ROC) analysis of biomarkers and metabolic ratios for discrimination of chronic migraine. (**A**) Receiver operating characteristic (ROC) curves demonstrating the discriminatory performance of individual hypoxia-related, endothelial, and arginine pathway biomarkers for distinguishing patients with chronic migraine from healthy controls. VEGF-A exhibited the highest classification performance (AUC = 0.973), followed by HIF-1α (AUC = 0.794). Ornithine, arginine, and citrulline showed lower discriminatory ability. (**B**) ROC curves demonstrating the discriminatory performance of ratio-based indices derived from arginine metabolism for distinguishing patients with chronic migraine from healthy controls. Among these parameters, the arginine-to-citrulline ratio exhibited the highest classification performance (AUC = 0.696).

Receiver operating characteristic (ROC) analyses were performed to evaluate the discriminatory performance of individual biomarkers for migraine status (Table 3, Figure 3A,B).

The dashed diagonal line represents the line of no discrimination (AUC = 0.5), corresponding to random classification performance.

VEGF-A demonstrated the highest discriminatory performance, with an AUC of 0.973 (95% CI, 0.941–1.000). At the optimal Youden threshold of 742.34 pg/mL, VEGF-A achieved a sensitivity of 96.4% and a specificity of 89.3%.

HIF-1α showed good discriminatory performance, with an AUC of 0.794 (95% CI, 0.678–0.910). At a threshold of 909.06 pg/mL, HIF-1α achieved a sensitivity of 96.4% and a specificity of 50.0%.

Among arginine pathway biomarkers, the arginine-to-citrulline ratio showed the highest classification performance (AUC 0.696, 95% CI 0.553–0.840), followed by ornithine (AUC 0.680, 95% CI 0.534–0.826). Citrulline (AUC 0.666), arginine-to-ornithine ratio (AUC 0.662), arginine (AUC 0.551), and citrulline-to-ornithine ratio (AUC 0.527) demonstrated limited discriminatory ability.

### 3.5. Multivariable Firth Logistic Regression Analyses

**Figure 4:**
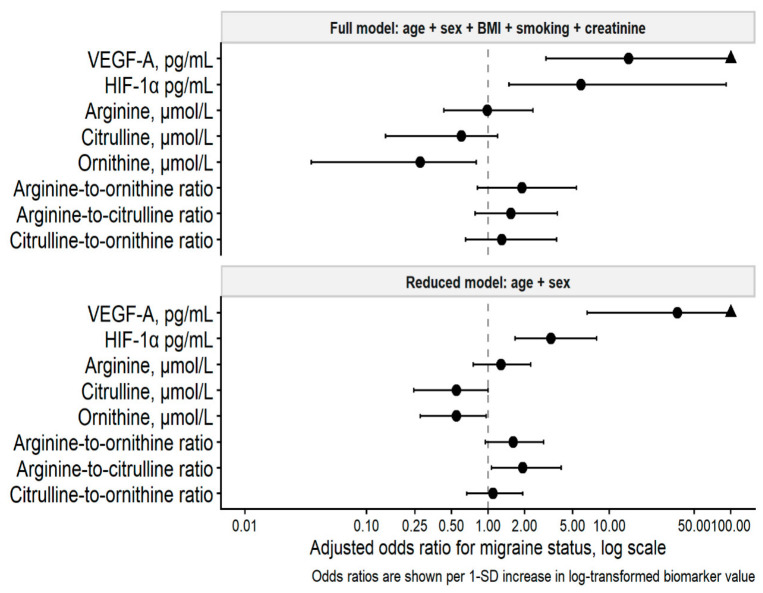
Adjusted Associations of Hypoxia-, Endothelial-, and Arginine Pathway Biomarkers with Chronic Migraine Status. Forest plots showing odds ratios (ORs) and 95% confidence intervals estimated using Firth penalized logistic regression models. The upper panel presents the fully adjusted model controlling for age, sex, body mass index (BMI), smoking status, and serum creatinine concentration. The lower panel presents the reduced model adjusted for age and sex only. Odds ratios are expressed per 1-standard-deviation increase in log-transformed biomarker values. The vertical dashed line indicates the null effect (OR = 1). Circles represent odds ratio estimates and horizontal lines indicate 95% confidence intervals. The triangle denotes estimates exceeding the plotting range (or extreme OR values, if applicable).

To determine whether biomarker associations were independent of potential confounding factors, Firth penalized logistic regression models were constructed adjusting for age, sex, body mass index, smoking status, and serum creatinine concentration (Figure 4).

In the fully adjusted model, VEGF-A, HIF-1α, and ornithine remained independently associated with migraine status after FDR correction. VEGF-A demonstrated the strongest association, with each one-standard-deviation increase in log-transformed VEGF-A associated with a 14.46-fold increase in the odds of migraine (adjusted OR = 14.46, 95% CI 3.00–4619.50; FDR-adjusted *p* < 0.001).

HIF-1α was also independently associated with migraine status (adjusted OR = 5.83, 95% CI 1.50–91.71; FDR-adjusted *p* = 0.027).

Ornithine demonstrated a significant inverse association with migraine (adjusted OR = 0.28, 95% CI 0.04–0.80; FDR-adjusted *p* = 0.037).

No independent associations were observed for arginine, citrulline, arginine-to-citrulline ratio, arginine-to-ornithine ratio, or citrulline-to-ornithine ratio after adjustment and correction for multiple testing.

VEGF-A, HIF-1α, and ornithine were the only biomarkers that remained independently associated with migraine status after multivariable adjustment and correction for multiple testing.

Additional exploratory analyses evaluating associations between biomarker levels and migraine severity measures (monthly migraine frequency, MIDAS grade, and VAS score) are presented in Supplementary Figure S1.

### 3.6. Exploratory Principal Component Analysis

Exploratory principal component analysis demonstrated partial separation between patients with chronic migraine and healthy controls. The first two principal components explained 43.0% of the total variance, with PC1 accounting for 25.0% and PC2 for 18.0%. Separation was driven primarily by PC1, with migraine cases clustering toward negative PC1 scores and controls toward positive PC1 scores. Variable loadings indicated that VEGF-A, HIF-1α, BMI, smoking status, urea, and creatinine contributed predominantly to the negative PC1 direction, whereas ornithine and citrulline loaded in the opposite direction. Overall, these findings suggest that the observed group differences reflect a multidimensional pattern involving hypoxia-related, endothelial, metabolic, and clinical features rather than alterations in a single biomarker (Supplementary Figure S2).

Additional exploratory machine learning analyses evaluating the combined discriminatory performance of biomarker and clinical variables are presented in Supplementary Figure S3.

## 4. Discussion

The present study investigated the relationship between hypoxia-related signaling, endothelial activation, and arginine metabolism in patients with chronic migraine. Three principal findings emerged. First, circulating concentrations of HIF-1α and VEGF-A were significantly elevated in patients with chronic migraine compared with healthy controls. Second, alterations in arginine pathway metabolism were observed, characterized by lower ornithine concentrations and a higher arginine-to-citrulline ratio. Third, VEGF-A, HIF-1α, and ornithine remained independently associated with migraine status after multivariable adjustment, whereas VEGF-A demonstrated excellent discriminatory performance in receiver operating characteristic analyses. Collectively, these findings are consistent with the hypothesis that hypoxia-responsive signaling, endothelial activation, and alterations in arginine metabolism may coexist in chronic migraine.

Migraine is increasingly recognized as a complex neurovascular disorder involving dynamic interactions among neuronal, vascular, inflammatory, and metabolic pathways rather than a purely vascular or neuronal condition [3,4,5,24,25]. Although the trigeminovascular system remains central to current concepts of migraine pathophysiology [26], growing evidence suggests that disturbances in cellular energy metabolism and tissue oxygen homeostasis may also contribute to disease susceptibility and attack generation [6,7,8,24,25]. Experimental hypoxia can provoke migraine-like attacks in susceptible individuals, while cortical spreading depolarization has been linked to profound metabolic stress and transient alterations in cerebral oxygen utilization [6,7,8]. Within this framework, hypoxia-responsive signaling pathways have emerged as biologically plausible contributors to migraine pathobiology.

One of the most important observations of the present study was the elevation of circulating HIF-1α concentrations in patients with chronic migraine. HIF-1α is the principal transcription factor regulating cellular adaptation to hypoxic stress and coordinates the expression of genes involved in angiogenesis, glucose utilization, oxidative stress responses, and vascular remodeling [9,10,27,28]. Under physiological conditions, HIF-1α is rapidly degraded; however, hypoxia, oxidative stress, and metabolic perturbations promote its stabilization and transcriptional activity [9,27,28]. Hypoxia and inflammatory signaling are also closely interconnected biological processes, providing a plausible framework through which hypoxia-responsive pathways may interact with neurovascular inflammation [29]. Compared with inflammatory mediators, neuropeptides, and vascular biomarkers, HIF-1α has received relatively limited attention in migraine biomarker research. Consequently, the present findings contribute to a growing body of evidence suggesting that hypoxia-responsive pathways warrant further investigation in chronic migraine. The higher HIF-1α concentrations observed in our cohort may reflect enhanced activation of adaptive responses to recurrent metabolic or hypoxic stress. Importantly, the association between HIF-1α and migraine status persisted after adjustment for demographic and clinical covariates, suggesting that the observed relationship is unlikely to be explained solely by differences in age, sex, smoking status, body composition, or renal function.

One of the notable findings of the present study was the marked elevation of VEGF-A concentrations. VEGF-A is a well-established downstream target of HIF-1α and plays a pivotal role in angiogenesis, endothelial activation, vascular permeability, and neurovascular remodeling [11,12,27]. Beyond its classical vascular actions, VEGF-A has been implicated in neuroinflammatory signaling and blood–brain barrier regulation, both of which have attracted increasing attention in migraine research [13,14,15,16,17,29]. Previous studies investigating circulating VEGF concentrations in migraine have reported heterogeneous findings, likely reflecting differences in migraine subtype, attack status, sample type, patient selection, and analytical methodology [16,17]. Our findings extend these observations by demonstrating markedly elevated VEGF-A concentrations, independent associations in multivariable models, and strong discriminatory performance in ROC analyses. Although direct comparisons should be interpreted cautiously, the magnitude of the observed VEGF-A signal suggests that endothelial activation may represent a particularly relevant biological feature of chronic migraine. Nevertheless, the strong diagnostic performance observed in the present cohort should be interpreted conservatively until external validation studies become available.

Alterations in arginine metabolism represented another important aspect of our findings. Nitric oxide has long been considered a key mediator in migraine pathophysiology, supported by experimental and clinical evidence demonstrating that nitric oxide donors can trigger migraine attacks and activate trigeminovascular pathways [18,19,20,26]. Arginine serves as the primary substrate for nitric oxide synthase, while citrulline and ornithine occupy central positions within interconnected metabolic pathways involving nitric oxide production, the urea cycle, and polyamine synthesis [21,30,31]. In the present study, lower ornithine concentrations and a higher arginine-to-citrulline ratio were observed in patients with chronic migraine. However, after multivariable adjustment, only ornithine remained independently associated with migraine status. Ornithine occupies a central metabolic position linking the urea cycle, arginase activity, and polyamine synthesis. Although the biological interpretation of reduced circulating ornithine remains uncertain, lower concentrations may reflect altered arginine utilization within interconnected nitric oxide-related pathways. Interestingly, arginine itself was not independently associated with migraine status, suggesting that pathway-level alterations may be more informative than isolated metabolite concentrations. Given that ornithine remained independently associated with migraine status after multivariable adjustment, this metabolite may warrant further investigation in future metabolic profiling studies. Within this framework, L-arginine and citrulline should be interpreted as functionally interconnected metabolites within the nitric oxide cycle, where citrulline serves as a precursor for de novo arginine regeneration, thereby contributing to sustained nitric oxide bioavailability. Therefore, circulating levels of these metabolites may reflect dynamic pathway activity rather than isolated substrate availability.

An important strength of the present study is the integrated evaluation of biomarkers representing hypoxia-responsive signaling, endothelial activation, and arginine metabolism within the same well-characterized chronic migraine cohort. Although these biological pathways have individually been investigated in previous migraine studies, most prior work has focused on vascular, inflammatory, or metabolic mechanisms in isolation [14,16,17]. By assessing these complementary domains within a single analytical framework, the present study provides a more integrated perspective on potential interactions among hypoxia-related, endothelial, and nitric oxide-associated metabolic processes in chronic migraine.

Recent reviews have emphasized that migraine is a complex and multifactorial disorder involving interactions among vascular, inflammatory, metabolic, and neurobiological mechanisms rather than isolated pathways. Emerging evidence from genomic, epigenetic, multi-omic, and biomarker-focused studies further suggests that integrated biomarker approaches may provide a more comprehensive understanding of migraine pathophysiology and facilitate the identification of clinically relevant biological signatures [32,33,34,35]. In this context, the present findings extend previous work by simultaneously evaluating hypoxia-responsive signaling (HIF-1α), endothelial activation (VEGF-A), and arginine-related metabolic pathways within the same chronic migraine cohort.

Additional support for such an integrated interpretation was provided by the correlation structure among the investigated biomarkers. HIF-1α and VEGF-A demonstrated a significant positive correlation, consistent with the established regulatory relationship between hypoxia-inducible signaling and VEGF expression [11,27]. Furthermore, VEGF-A showed inverse associations with ornithine, while several arginine pathway metabolites demonstrated coherent internal relationships. Although causality cannot be inferred from these cross-sectional data, the overall pattern is compatible with the possibility that hypoxia-responsive signaling, endothelial activation, and nitric oxide-related metabolic pathways are interconnected components of a broader biological response associated with chronic migraine.

The clinical implications of these findings should be interpreted cautiously. At present, migraine remains a clinically diagnosed disorder, and no circulating biomarker has been incorporated into routine diagnostic practice [3,17,24]. Nevertheless, objective biomarkers may eventually contribute to patient stratification, mechanistic phenotyping, and treatment-response studies. In this regard, the strong performance of VEGF-A and the independent associations observed for VEGF-A, HIF-1α, and ornithine warrant further evaluation in larger and independent populations. Whether these biomarkers reflect disease susceptibility, disease activity, attack frequency, or compensatory biological responses remains to be determined.

Several limitations merit consideration. First, the study was conducted at a single center and included a relatively modest sample size. Second, the cross-sectional design precludes conclusions regarding temporal relationships or causality. Third, biomarker measurements were obtained at a single time point and may not capture dynamic fluctuations across the migraine cycle. Fourth, patients with chronic migraine exhibited higher body mass index and smoking prevalence than healthy controls. Although multivariable adjustment was performed, residual confounding cannot be completely excluded. Fifth, post hoc analyses indicated limited statistical power for several moderate arginine-pathway effects, and therefore negative findings should be interpreted cautiously. In addition, none of the correlations with clinical severity measures (MIDAS and VAS scores) remained significant after correction for multiple comparisons, limiting conclusions regarding disease activity and the potential clinical utility of these biomarkers as indicators of migraine severity. Finally, external validation was not available, and therefore the observed diagnostic performance estimates should be considered preliminary and exploratory.

Methodological strengths of the study include the use of standardized fasting sample collection, LC–MS/MS-based amino acid quantification, and comprehensive multivariable analyses incorporating correlation analyses, ROC analyses, and Firth penalized logistic regression models. All participants fulfilled ICHD-3 diagnostic criteria for chronic migraine [23], blood samples were collected under standardized fasting conditions, and amino acid quantification was performed using LC–MS/MS methodology. Furthermore, the combined assessment of HIF-1α, VEGF-A, arginine pathway metabolites, correlation analyses, receiver operating characteristic analyses, and multivariable Firth regression models provided a comprehensive evaluation of hypoxia-related, endothelial, and metabolic biomarkers within the same study population.

## 5. Conclusions

In conclusion, patients with chronic migraine exhibited elevated circulating HIF-1α and VEGF-A concentrations together with alterations in arginine pathway metabolism. VEGF-A, HIF-1α, and ornithine remained independently associated with migraine status, while VEGF-A demonstrated excellent discriminatory performance in this cohort. Although the present findings do not establish causal relationships, they are consistent with the concept that hypoxia-responsive signaling, endothelial activation, and metabolic alterations may represent interconnected biological processes associated with chronic migraine. Further longitudinal and mechanistic studies are warranted to clarify the biological significance of these observations and to determine whether these pathways may contribute to future biomarker development in migraine.

## Figures and Tables

**Table 1:** Demographic, Clinical, and Laboratory Characteristics of Patients with Chronic Migraine and Healthy Controls.

Variable	Study Group			*p*-Value ^2^
	Overall N = 56 ^1^	Non-Migraine N = 28 ^1^	Migraine N = 28 ^1^	
**Age, years**	40.00 (30.50, 48.00)	39.50 (31.50, 48.50)	40.00 (29.50, 48.00)	>0.9
**Sex**				0.2
Female	39 (69.6%)	17 (60.7%)	22 (78.6%)	
Male	17 (30.4%)	11 (39.3%)	6 (21.4%)	
**Smoking status**				0.02 *
No	37 (66.1%)	23 (82.1%)	14 (50.0%)	
Yes	19 (33.9%)	5 (17.9%)	14 (50.0%)	
**Body mass index, kg/m^2^**	21.00 (19.00, 23.00)	19.00 (18.25, 20.50)	23.00 (21.00, 24.00)	<0.001 *
**Urea, mg/dL**	27.00 (24.00, 28.00)	27.00 (26.00, 28.00)	24.00 (18.50, 35.50)	0.5
**Creatinine, mg/dL**	0.60 (0.60, 0.70)	0.60 (0.60, 0.70)	0.60 (0.50, 0.75)	0.7

^1^ Median (Q1, Q3); n (%). ^2^ Wilcoxon rank-sum test for continuous variables; Fisher’s exact test for categorical variables. Continuous variables are presented as median (IQR). Categorical variables are presented as n (%). * *p* < 0.05 was considered statistically significant.

**Table 2:** Comparison of Hypoxia-Related, Endothelial, and Arginine Pathway Biomarkers between Patients with Chronic Migraine and Healthy Controls.

Biomarker	Healthy Controls, Median (IQR)	Chronic Migraine, Median (IQR)	Median Difference	Hodges–Lehmann Difference (95% CI)	Rank-Biserial r	SMD (Log Scale)	Raw *p*	FDR-Adjusted *p*
VEGF-A, pg/mL	279.41 (226.29–456.71)	1494.00 (1181.50–1776.71)	1214.59	1076.66 (873.33 to 1290.83)	0.946	2.936	<0.001	<0.001
HIF-1α, pg/mL	942.08 (710.94–1374.62)	1693.96 (1172.74–2360.00)	751.88	630.19 (307.54 to 1083.02)	0.588	1.162	<0.001	0.001
Arginine-to-citrulline ratio	1.19 (1.01–1.60)	1.60 (1.42–1.96)	0.41	0.41 (0.09 to 0.67)	0.393	0.602	0.010	0.032
Ornithine, µmol/L	102.60 (92.87–117.22)	81.10 (74.82–104.12)	−21.50	−16.88 (−29.96 to −4.07)	−0.360	−0.644	0.020	0.043
Citrulline, µmol/L	33.53 (28.08–38.96)	28.25 (23.33–35.10)	−5.28	−4.65 (−9.74 to −0.33)	−0.332	−0.582	0.030	0.051
Arginine-to-ornithine ratio	0.45 (0.30–0.58)	0.57 (0.41–0.76)	0.12	0.14 (0.01 to 0.27)	0.324	0.507	0.040	0.051
Arginine, µmol/L	39.72 (33.02–56.45)	45.33 (34.95–53.42)	5.61	2.63 (−5.33 to 12.03)	0.102	0.197	0.520	0.590
Citrulline-to-ornithine ratio	0.32 (0.26–0.39)	0.34 (0.26–0.45)	0.02	0.01 (−0.05 to 0.08)	0.054	0.125	0.740	0.740

Note: Median differences and Hodges–Lehmann estimates are reported as chronic migraine minus healthy control values. Between-group comparisons were performed using Wilcoxon rank-sum tests. Raw *p*-values were adjusted for multiple testing using the Benjamini–Hochberg false discovery rate (FDR) procedure. SMD values were calculated using log-transformed biomarker concentrations. Abbreviations: CI, confidence interval; FDR, false discovery rate; HIF-1α, hypoxia-inducible factor-1 alpha; SMD, standardized mean difference; VEGF-A, vascular endothelial growth factor A.

**Table 3:** Receiver Operating Characteristic Analysis of Hypoxia-Related, Endothelial, and Arginine Pathway Biomarkers for the Discrimination of Chronic Migraine.

Biomarker	AUC	95% CI	Optimal Youden Threshold	Sensitivity	Specificity
VEGF-A, pg/mL	0.973	0.941–1.000	742.34	0.964	0.893
HIF-1α, pg/mL	0.794	0.678–0.910	909.06	0.964	0.500
Arginine-to-citrulline ratio	0.696	0.553–0.840	1.379	0.786	0.643
Ornithine, µmol/L	0.680	0.534–0.826	84.20	0.571	0.857
Citrulline, µmol/L	0.666	0.523–0.809	37.41	0.893	0.393
Arginine-to-ornithine ratio	0.662	0.515–0.809	0.532	0.643	0.714
Arginine, µmol/L	0.551	0.394–0.708	42.07	0.714	0.536
Citrulline-to-ornithine ratio	0.527	0.369–0.685	0.325	0.607	0.536

Note: ROC analyses were performed to evaluate the discriminatory ability of individual biomarkers for distinguishing patients with chronic migraine from healthy controls. Optimal thresholds were determined using the Youden index. Sensitivity and specificity are reported at the corresponding optimal cutoff values. Abbreviations: AUC, area under the receiver operating characteristic curve; CI, confidence interval; HIF-1α, hypoxia-inducible factor-1 alpha; VEGF-A, vascular endothelial growth factor A.

## Data Availability

The data presented in this study are available from the corresponding author upon reasonable request.

## Supplementary Materials

The following supporting information can be downloaded at: https://www.mdpi.com/article/10.3390/biomedicines14071458/s1. Supplementary Table S1. Distributional characteristics and normality assessment of biomarkers stratified by study group. Supplementary Table S2. Geometric mean summaries of hypoxia-related, endothelial, and arginine pathway biomarkers. Figure S1. Exploratory associations between circulating biomarkers and migraine severity measures in patients with chronic migraine. Figure S2. Principal component analysis (PCA) of combined biomarker and clinical variables in migraine and non-migraine participants. Figure S3. Exploratory machine learning analyses comparing biomarker-only and combined biomarker-clinical models.

## Abbreviations

The following abbreviations are used in this manuscript:

AUC	Area Under the Curve
BMI	Body Mass Index
CI	Confidence Interval
ELISA	Enzyme-Linked Immunosorbent Assay
FDR	False Discovery Rate
HIF-1α	Hypoxia-Inducible Factor-1 Alpha
ICHD-3	International Classification of Headache Disorders, 3rd Edition
IQR	Interquartile Range
LC–MS/MS	Liquid Chromatography–Tandem Mass Spectrometry
MIDAS	Migraine Disability Assessment Scale
MRM	Multiple Reaction Monitoring
NO	Nitric Oxide
OR	Odds Ratio
PCA	Principal Component Analysis
ROC	Receiver Operating Characteristic
SD	Standard Deviation
SMD	Standardized Mean Difference
VAS	Visual Analog Scale
VEGF-A	Vascular Endothelial Growth Factor A
