# Supplementary material for: Evidence of Hypoxia Signaling and Endothelial Activation in Migraine: Relationships Between HIF-1α, VEGF-A, and Arginine Metabolism
Source: Biomedicines. 2026 Jun 27;14(7):1458. doi: 10.3390/biomedicines14071458 (PMC13405583; doi:10.3390/biomedicines14071458)
Supplement: Supplementary file 1 [file biomedicines-14-01458-s001.zip › biomedicines-4387190-supplementary.pdf]

Supplementary Table S1. Distributional characteristics and normality assessment of biomarkers stratified by study group.

Biomarker	Group	n	Mean	SD	Median	Q1	Q3	Min	Max	Shapiro–Wilk p	Distribution
Arginine-to-citrulline ratio	Non-migraine	28	1.437	0.752	1.190	1.008	1.601	0.562	3.988	<0.001	Non-normally distributed
Arginine-to-citrulline ratio	Migraine	28	1.884	1.019	1.599	1.416	1.960	0.836	6.173	<0.001	Non-normally distributed
Arginine-to-ornithine ratio	Non-migraine	28	0.485	0.261	0.451	0.300	0.577	0.137	1.061	0.010	Non-normally distributed
Arginine-to-ornithine ratio	Migraine	28	0.607	0.248	0.574	0.407	0.763	0.175	1.128	0.842	Approximately normally distributed
Arginine (μmol/L)	Non-migraine	28	45.922	18.802	39.716	33.015	56.451	21.323	99.779	0.010	Non-normally distributed
Arginine (μmol/L)	Migraine	28	47.906	15.249	45.330	34.952	53.423	23.922	92.293	0.081	Approximately normally distributed
Citrulline-to-ornithine ratio	Non-migraine	28	0.338	0.095	0.320	0.264	0.386	0.223	0.578	0.020	Non-normally distributed
Citrulline-to-ornithine ratio	Migraine	28	0.364	0.193	0.342	0.264	0.450	0.089	1.108	<0.001	Non-normally distributed
Citrulline (μmol/L)	Non-migraine	28	34.259	9.657	33.531	28.076	38.960	14.033	64.289	0.100	Approximately normally distributed
Citrulline (μmol/L)	Migraine	28	28.616	9.175	28.254	23.328	35.103	5.329	51.800	0.466	Approximately normally distributed
HIF-1α (pg/mL)	Non-migraine	28	1111.078	545.270	942.075	710.942	1374.625	487.360	2836.420	0.006	Non-normally distributed
HIF-1α (pg/mL)	Migraine	28	1986.010	1118.199	1693.960	1172.740	2359.997	843.960	5479.810	0.001	Non-normally distributed
Ornithine (μmol/L)	Non-migraine	28	104.158	25.845	102.603	92.873	117.219	52.740	172.625	0.399	Approximately normally distributed
Ornithine (μmol/L)	Migraine	28	87.542	28.434	81.101	74.825	104.123	32.308	144.211	0.459	Approximately normally distributed
VEGF-A (pg/mL)	Non-migraine	28	395.845	260.688	279.415	226.295	456.707	166.920	1160.250	<0.001	Non-normally distributed
VEGF-A (pg/mL)	Migraine	28	1418.195	423.052	1494.000	1181.497	1776.710	373.580	1967.750	0.016	Non-normally distributed

Supplementary Table S2. Geometric mean summaries of hypoxia-related, endothelial, and arginine pathway biomarkers.

Biomarker	Control geometric mean (GSD)	95% CI	Median (Q1–Q3)	Chronic migraine geometric mean (GSD)	95% CI	Median (Q1–Q3)
Arginine-to-citrulline ratio	1.29 (1.57)	1.10–1.53	1.19 (1.01–1.60)	1.72 (1.51)	1.47–2.00	1.60 (1.42–1.96)
Arginine-to-ornithine ratio	0.42 (1.72)	0.35–0.52	0.45 (0.30–0.58)	0.55 (1.59)	0.47–0.66	0.57 (0.41–0.76)
Arginine (μmol/L)	42.68 (1.47)	37.02–49.02	39.72 (33.02–56.45)	45.74 (1.36)	40.81–51.28	45.33 (34.95–53.42)
Citrulline-to-ornithine ratio	0.33 (1.30)	0.30–0.36	0.32 (0.26–0.39)	0.32 (1.67)	0.27–0.39	0.34 (0.26–0.45)
Citrulline (μmol/L)	32.98 (1.33)	29.66–36.66	33.53 (28.08–38.96)	26.68 (1.54)	22.73–31.31	28.25 (23.33–35.10)
HIF-1α (pg/mL)	1002.04 (1.58)	846.36–1186.36	942.08 (710.94–1374.63)	1747.01 (1.65)	1451.63–2102.50	1693.96 (1172.74–2360.00)
Ornithine (μmol/L)	101.03 (1.29)	91.90–111.07	102.60 (92.87–117.22)	82.72 (1.43)	72.47–94.41	81.10 (74.83–104.12)
VEGF-A (pg/mL)	336.94 (1.72)	275.48–412.11	279.42 (226.30–456.71)	1337.69 (1.47)	1161.32–1540.84	1494.00 (1181.50–1776.71)

S3. Simulation-based power and sensitivity analyses

Simulation-based power analyses indicated that the study was well powered to detect large effects in VEGF-A and HIF-1α, whereas power was more limited for smaller effect sizes observed in arginine pathway biomarkers. For group comparisons based on log-transformed biomarker values, VEGF-A (d = 2.94) and HIF-1α (d = 1.16) demonstrated large effect sizes, while ornithine (d = −0.644), arginine-to-citrulline ratio (d = 0.602), citrulline (d = −0.582), and arginine-to-ornithine ratio (d = 0.507) showed moderate effect sizes.

With 28 participants per group, the minimum detectable standardized mean difference was 0.762 at α = 0.05 and 0.942 at α = 0.01, indicating that the study was primarily powered to detect moderate-to-large effects.

ROC-based sensitivity analyses further supported strong performance for VEGF-A (AUC = 0.973; power = 1.000) and HIF-1α (AUC = 0.794; power = 0.987), whereas arginine pathway markers showed lower discriminatory power. The minimum detectable AUC with 80% power was 0.708.

In multivariable Firth logistic regression models, simulation-based power was highest for VEGF-A, reaching 98.3% at α = 0.05 and 85.5% under the multiplicity-adjusted threshold (α = 0.00625). HIF-1α demonstrated adequate power at α = 0.05 (78.5%) but reduced power after correction (44.1%). Ornithine showed moderate-to-low power (65.7% at α = 0.05; 29.1% at α = 0.00625), while remaining arginine pathway biomarkers were underpowered in adjusted models.

S4. Exploratory associations with migraine severity

Among patients with migraine, exploratory correlation analyses indicated that monthly migraine frequency showed the strongest associations with biomarker levels (Supplementary Figure S1). Ornithine was inversely correlated with frequency (ϱ = −0.467; raw p = 0.012; FDR-adjusted p = 0.081), whereas the

arginine-to-ornithine ratio showed a positive correlation (ϱ = 0.437; raw p = 0.020; FDR-adjusted p = 0.081). VEGF-A also demonstrated a moderate positive association with monthly frequency (ϱ = 0.389; raw p = 0.041; FDR-adjusted p = 0.109).

None of these associations remained statistically significant after correction for multiple testing and should therefore be interpreted as exploratory.

No significant associations after FDR correction were observed for MIDAS grade or VAS score. For MIDAS grade, the strongest correlations were observed for VEGF-A (ϱ = 0.350; FDR-adjusted p = 0.368) and ornithine (ϱ = −0.312; FDR-adjusted p = 0.368). For VAS score, the strongest association was observed for arginine (ϱ = 0.356; FDR-adjusted p = 0.503) (Supplementary Figure S1).

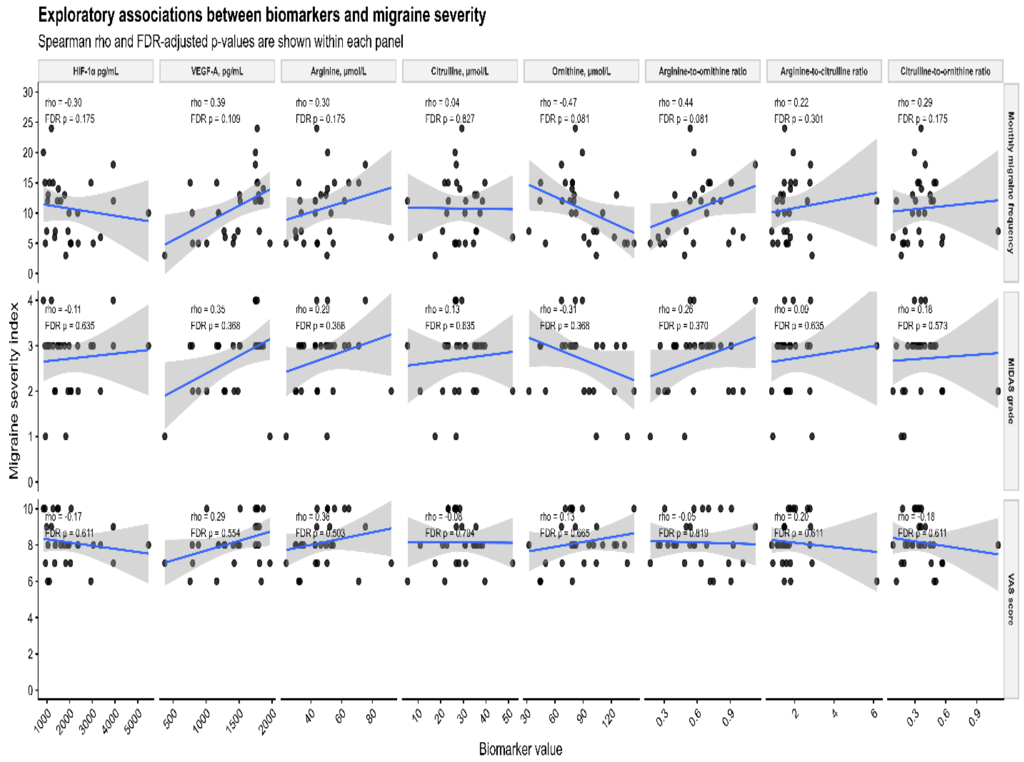

Figure S1. Exploratory associations between circulating biomarkers and migraine severity measures in patients with chronic migraine. Scatterplots showing Spearman rank correlations between biomarker levels (HIF-1α, VEGF-A, arginine, citrulline, ornithine, arginine-to-ornithine ratio, arginine-to-citrulline ratio, and citrulline-to-ornithine ratio) and monthly migraine frequency, MIDAS grade, and VAS score. Blue lines represent fitted linear trend lines with 95% confidence intervals (shaded areas). Spearman correlation coefficients (ϱ) and false discovery rate (FDR)-adjusted p-values are displayed within each panel. None of the observed associations remained statistically significant after correction for multiple testing and should therefore be interpreted as exploratory.

S5. Principal component analysis: Principal component analysis of the combined biomarker and clinical dataset showed partial separation between patients with migraine and non-migraine controls. The first two principal components explained 39.3% of the total variance, with PC1 accounting for 22.8% and PC2 for 16.5%. Separation was driven primarily by PC1, with non-migraine controls clustering toward positive PC1 scores and migraine cases toward negative PC1 scores. Group centroid analysis supported this pattern, with mean PC1 scores of 1.249 ± 0.873 in controls and −1.249 ± 1.048 in migraine cases. PC2 showed less distinct between-group separation but greater dispersion within the migraine group, suggesting heterogeneity within the migraine group.

The PCA loadings indicated that VEGF-A, HIF-1α, BMI, smoking, urea, and creatinine primarily loaded in the negative PC1 direction, whereas ornithine, citrulline, and male sex loaded in the positive PC1 direction. Thus, the observed group separation appeared to reflect a multivariable pattern encompassing endothelial, hypoxia-related, metabolic, and clinical features rather than a single marker. Although the confidence ellipses overlapped at the center, the overall distribution suggested a shift in the combined biomarker-clinical profile among migraine cases (Figure S2).

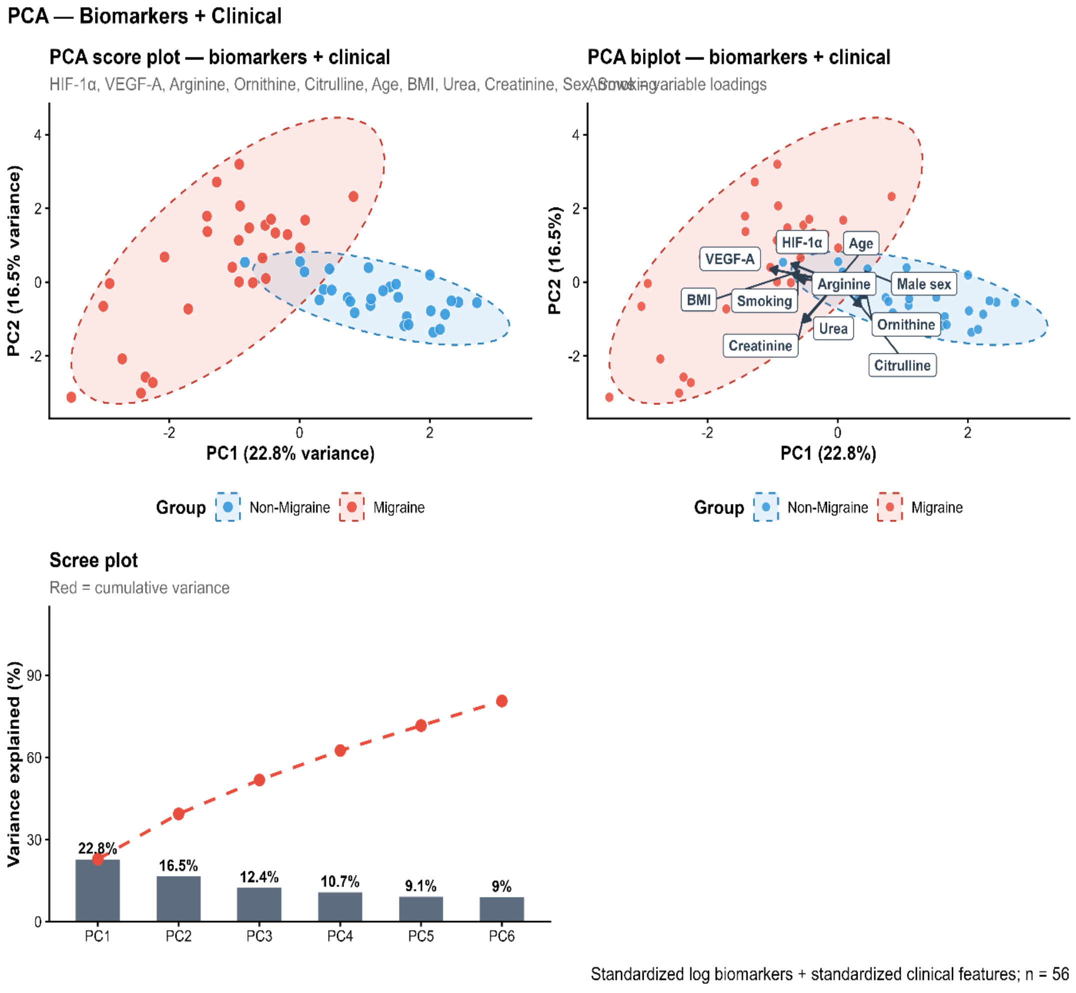

Figure S2. Principal component analysis (PCA) of combined biomarker and clinical variables in migraine and non-migraine participants. Principal component analysis was performed using standardized biomarker (HIF-1α, VEGF-A, arginine, ornithine, and citrulline) and clinical variables (age, BMI, urea, creatinine, sex, and smoking status). The PCA score plot (left) shows the distribution of migraine and non-migraine participants according to the first two principal components, with dashed ellipses representing the 95% confidence regions for each group. The PCA biplot (right) displays variable loadings, illustrating the contribution of individual biomarkers and clinical variables to group separation. The scree plot (bottom) shows the proportion of variance explained by the first six principal components and the cumulative explained variance. The first two principal

components accounted for 43.0% of the total variance and demonstrated partial separation between migraine and non-migraine groups, suggesting differences in their combined biomarker-clinical profiles.

S6. Supplementary Machine Learning-Based Results:

Exploratory machine learning analyses were performed to assess whether combined biomarker and clinical features improved discrimination between migraine and non-migraine participants. Linear discriminant analysis (LDA) and support vector machine (SVM) models were evaluated using leave-one-out cross-validation (LOOCV). Models incorporating both biomarkers and clinical variables demonstrated improved classification performance compared with biomarker-only models. LDA classification accuracy increased from 89.3% to 94.6%, while the SVM area under the receiver operating characteristic curve (AUC) increased from 0.941 to 0.981 when clinical variables were included. These findings suggest that combined biomarker-clinical profiles may provide greater discriminatory information than biomarkers alone. Given the modest sample size and exploratory nature of these analyses, the results should be interpreted cautiously and require validation in independent cohorts.

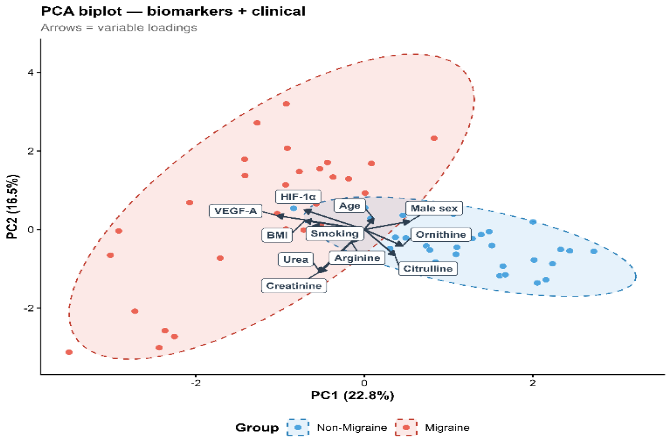

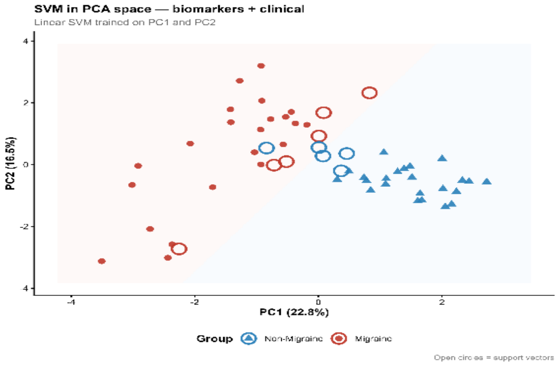

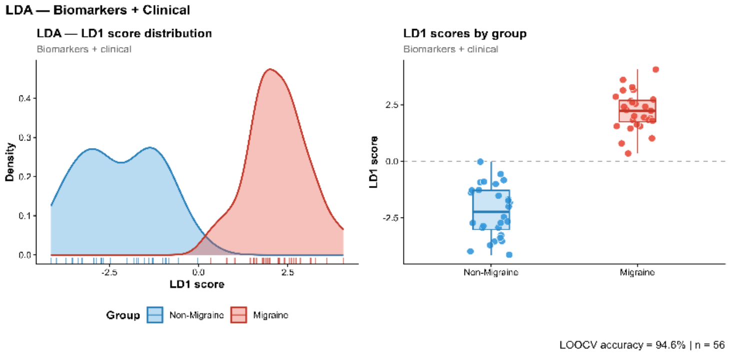

Model comparison summary

Biomarkers-only vs Biomarkers + Clinical | n = 56

Method	Result
**A — Biomarkers only**	
LDA (LOOCV)	89.3%
SVM in PCA space (LOOCV AUC)	0.941
PCA variance (PC1+PC2)	60.8%
**B — Biomarkers + Clinical**	
LDA (LOOCV)	94.6%
SVM in PCA space (LOOCV AUC)	0.981
PCA variance (PC1+PC2)	39.3%

Figure S3. Exploratory machine learning analyses comparing biomarker-only and combined biomarker-clinical models.

Principal component analysis (PCA), support vector machine (SVM), and linear discriminant analysis (LDA) were used to evaluate discrimination between migraine and non-migraine participants. The PCA biplot illustrates the distribution of participants and variable loadings in the reduced-dimensional feature space. The SVM plot shows classification boundaries in the PCA space using leave-one-out cross-validation (LOOCV). LDA plots display the distribution of discriminant scores and group separation. The summary table compares classification performance between biomarker-only models and models incorporating both biomarkers and clinical variables. Inclusion of clinical variables improved classification performance, yielding an LDA accuracy of 94.6% and an SVM AUC of 0.981. These analyses were exploratory and are not intended as validated diagnostic models.
